# Evaluating the complete (44-item), short (20-item) and ultra-short (10-item) versions of the Big Five Inventory (BFI) in the Brazilian population

**DOI:** 10.1038/s41598-023-34504-1

**Published:** 2023-05-05

**Authors:** Raul Costa Mastrascusa, Matheus Loli de Oliveira Fenili Antunes, Nathalia Saraiva de Albuquerque, Sara Luísa Virissimo, Marcela Foletto Moura, Bibiana Vieira Marques Motta, Wagner de Lara Machado, Carmen Moret-Tatay, Tatiana Quarti Irigaray

**Affiliations:** 1 Pontifícia Universidade Católica do Rio Grande do Sul, Porto Alegre, Brazil; 2 Universidad Católica de Valencia San Vicente Mártir, Valencia, Spain

**Keywords:** Human behaviour, Psychology, Health care

## Abstract

The Big Five Inventory (BFI) is an instrument designed to assess the personality of individuals aged 18 and above. The original version consists of 44 items divided into five sub-scales representing each of the five personality factors: agreeableness, neuroticism, conscientiousness, openness, and extraversion. The main purpose of this study was to assess the factorial structure of the 44-item BFI and the reliability of two shorter versions with 20 and 10 items. The study also aimed to present normative data for interpreting scores from the short and ultrashort versions of the BFI for the Brazilian population. A total of 3565 individuals with a mean age of 33.3 years (SD = 13.0) from all Brazilian states participated in the study, with 44.2% from the State of Rio Grande do Sul. Participants completed a sociodemographic questionnaire and the BFI. Confirmatory factor analysis showed poor adaptation of the original 44-item model, but the short and ultrashort versions with 20 and 10 items respectively had good adaptation indexes and reliability, with Omega coefficients above 0.70. Normative data for the shorter versions were presented using mean, standard deviation, and percentiles (lower, medium, and higher). The study concluded that the short and ultrashort versions of the BFI have good reliability and can be used in surveys requiring a brief personality assessment.

## Introduction

The term ‘personality’ is widely discussed by psychologists, so much so that there is not a single theory, but rather a combination of clinical and scientific observations^1^. One of the most recognized theories for studying personality is the five-factor theory, which defines personality according to five factors: openness, conscientiousness, extraversion, agreeableness, and neuroticism^2^. Openness refers to complexity, being open to new experiences, and depth. Conscientiousness is associated with one controlling one’s impulses, and individuals with high scores on this factor tend to be reliable, organized, and responsible. Extraversion is related to activity, energy, expressiveness, sociability, dominance, and enthusiasm. Agreeableness indicates affection, cooperativeness, and proper kindness. Finally, neuroticism refers to emotional instability, and individuals with a high score on this factor are generally sensitive, tense, and preoccupied^3^.

There are several instruments based in the big five personality factors model, such as the Revised NEO Personality Instrument (NEO PI-R)^4^, the Trait Descriptive Adjective (TDA)^5^, the International Personality Item Pool (IPIP)^5^, and the Big Five Inventory (BFI)^6^. Among these, the standout would be the BFI, a globally recognized instrument that, in addition to being free, can be used by professionals other than psychologists^7^, hence being a widely used instrument for survey and research purposes. The main goal of the BFI is to assess the personality of individuals over the age of 18. This instrument consists of 44 items divided into five sub-scales represented by each one of the five personality factors^6^.

The original version of the BFI (with 44 items) has already been translated and validated for many countries, such as Italy^8^, Denmark^9^, the Netherlands^10^, and Germany^11^. In Brazil, the original version of the instrument was adjusted and validated by Andrade^12^, and more recently by Junior et al.^7^.

In countries such as France, the United States and Germany, there is a short version of the BFI with 10 items, the performance of which proved to be equivalent to that of the original version^13,14^. In Brazil, Junior et al.^7^ validated a shorter version, featuring 25 items, maintaining the structure with the five factors, which provide support to the instrument’s original theory.

In the context of surveys, there is an increasing need to collect information in a short period of time, which highlights the importance of instruments that can be filled out quickly and have good reliability. Thus, considering the potential use of the BFI in surveys and the need for an instrument that can assess personalities in a quick way and be reliable, the aim of this study was to assess the factorial structure of the BFI 44 items and analyze the reliability of the short and ultrashort version of the BFI, with 20 and 10 items, respectively. Moreover, we sought to present normative data that refer to the interpretation of test scores for the Brazilian population of the BFI (Big Five Inventory).

## Methods

### Participants

Participants of the present study were 3565 adults, mean age of 33.3 (SD = 13.0), 2788 (71.5%) females, mainly with uncompleted major degrees (1212, 34%) or postgraduate studies (1086, 30.5%). Participants were from all geographic regions of Brazil, mainly Rio Grande do Sul (1553, 44.2%) or São Paulo (687, 19.6%). In terms of occupation, 54.7% were actively working; 8.3% unemployed, 4.3% retired, 29.1% students and 3.6% indicated “others”. With regards to the marital status, 35% were married, 5.6% divorced, 1.2% widowed and 58.2% single. A total of 73% of the participants did not have children. Regarding their living situation, 85.9% lived with family members or friends, whereas 14.1% lived alone.

### Instruments

Questionnaire of demographic data: that aimed the collection of the sample’s sociodemographic characterization data, including factors such as age, sex, state, marital status, education, professional status and housing.

*Big Five Inventory*—BFI (John et al., 1991). BFI aimed to evaluate the personality dimensions through 44 items, structured by simple sentences, and rated in the Likert scale of 5 points, ranging from 1 (totally disagree) to 5 (totally agree)^6^. The BFI has been translated and validated for the Brazilian population, showing adequate psychometric properties and coefficients of Cronbach’s Alpha of 0.65, 0.75, 0.75, 0.64 and 0.69 respectively for the five factors of “Openness”, “Neuroticism”, “Extraversion”, “Conscientiousness” and “Agreeableness”. The Portuguese version of the BFI applied in this study was that of Andrade^12^.

### Ethics and procedures

Data collection was carried out online, through an electronic questionnaire on the Qualtrics platform, and took place between the beginning of September and the end of November 2020. Participants were recruited through dissemination on social networks (invitations on Facebook, Instagram, among others) and e-mails sent to universities in different regions of Brazil.

This study was approved by the Research Ethics Committee of the Pontifícia Universidade Católica do Rio Grande do Sul (PUCRS) under number: 36641120.3.0000.5336. All participants voluntarily agreed to participate in the research by agreeing to a Free and Informed Consent Term (FICT).

### Design and data analysis

This is a cross-sectional study. Data analysis was conducted using R environment (R Core Team, 2020), and lavaan package^15^. To examine internal consistency, McDonald’s ω was employed as it is recommended when there are multiple sources of measurement error, more than two items in the scale, or nonlinear relationships between the items^16^. Confirmatory factor analysis was performed to investigate the original 44 items and five factors model fit to the data. Further confirmatory factor analysis was used to test a concurrent, refined model with the four best indicators per factor. An additional ultra-short model with ten items and five factors, for survey purposes only, was analyzed by the means of exploratory factor analysis, due to identification criteria.

Estimate methods were Diagonally Weighted Least Squares (DWLS) for confirmatory factor analysis and minimum rank factor analysis for exploratory factor analysis, using a polychoric correlation matrix of the items. To assess the fit of the models to the data, we consider the use of Comparative Fit Index, Tucker-Lewis Index (CFI and TLI, > 0.95) and Root Mean Square Error of Approximation (RMSEA, < 0.06 or 0.008 with 90% confidence interval) (Hair et al., 2019). Reliability was assessed with Omega coefficient, with expected values equal or higher than 0.70. Bivariate correlation analysis was carried between personality factors of short (20 items) and ultra-short (10 items), to assess validity of the ultra-short version.

### Institutional Review Board Statement

The study was conducted in accordance with the Declaration of Helsinki, and approved by the instituional Ethics Committee.

### Informed consent

Written informed consent has been obtained from the participants.

## Results

The original model of BFI, with 44 items and five factors, showed poor fit to the data according to fit indices: x^2^ (892) = 26,505.9, CFI/TLI = 0.87/0.86, RMSEA = 0.09 (0.09–0.09, 90% CI). The ultrashort version was based on the study of Rammstedt e John (2007), while the short version was derived from empirical information, i.e., the four higher loading items from each factor. We do not examined the BFI-10 item structure due to low loadings of those items in the short version. Respecified model with best four indicators per factor presented well-fit to the data, according to fit indices: x^2^(160) = 3443.9, CFI/TLI = 0.96/0.95, RMSEA = 0.08 (0.07–0.08, 90% CI). Item factor loadings and factor reliability in short (20 items) and ultra-short (10 items) versions of BFI are presented in Table 1. The factor loadings was determined by CFA for short and EFA for ultrashort versions. The factor loadings was determined by the Inter-factor correlation was presented in Factor Correlation Table 2. In order to demonstrate the validity of the ultra-short version of the BFI, we presented the bivariate correlation between five personality factors of both versions of the scale, short and ultra-short, the normative data are presented in the Table 3 to indicate the typical or average performance that is observed among participants.

**Table 1:** Factor Reliability Index Table. Factor loadings of items and reliability in short (20 items) and ultra-short (10 items) versions of BFI.

Personality factor	Items factor loading	Factor reliability (Omega) short/ultra-short
Openness	BFI9* = 0.92/0.82	ω = 0.82/0.88
	BFI11* = 0.86/0.88	
	BFI33 = 0.54	
	BFI39 = 0.56	
Conscientiousness	BFI6 = 0.64	ω = 0.76/0.72
	BFI19 = 0.57	
	BFI20* = 0.70/0.79	
	BFI31* = 0.78/56	
Agreeableness	BFI8* = 0.78/0.87	ω = 0.83/81
	BFI15* = 0.83/0.62	
	BFI18 = 0.72	
	BFI27 = 0.67	
Neuroticism	BFI7 = 0.75	ω = 0.83/0.84
	BFI14 = 0.67	
	BFI34* = 0.79/0.88	
	BFI36* = 0.82/0.76	
Extraversion	BFI1* = 0.82/0.85	ω = 0.86/0.87
	BFI26* = 0.90/0.83	
	BFI29 = 0.75	
	BFI42 = 0.68	

*Items selected for the ultra-short version. For those items, the first factor loading is from short (20 items) versions and the second factor load is from ultra-short (10 items) versions.

**Table 2:** Inter factor correlations of short (20 items) and ultra-short (10 items) versions of BFI.

Personality factor	Openness	Extraversion	Neuroticism	Agreeableness	Conscientiousness
Openness	0.89	0.29	− 0.13	0.23	0.31
Extraversion	0.34	0.93	− 0.14	0.32	0.26
Neuroticism	− 0.16	− 0.17	0.91	− 0.12	− 0.15
Agreeableness	0.26	0.39	− 0.15	0.90	0.36
Conscientiousness	0.38	0.32	− 0.17	0.44	0.87

**Table 3:** Normative data table to reflect the typical or average performance in the population under study.

Classification	Percentile	Open 20	Open 10	Cons 20	Cons 10	Soc 20	Soc 10	Neu 20	Neu 10	Ext 20	Ext 10
Lower	0	0.0	0.0	0.0	0.0	0.25	0.0	0.0	0.0	0.0	0.0
	25th	2.5	2.0	2.5	3.5	3.5	3.0	1.06	1.5	7.75	2.0
Medium	50th	3.0	3.0	3.25	4.0	4.25	4.0	2.0	2.5	2.75	3.0
Higher	75th	4.0	4.0	4.0	5.0	5.0	5.0	2.75	3.0	3.75	4.0
	100th	5.0	5.0	5.0	5.0	5.0	5.0	5.0	5.0	5.0	5.0
	Mean(SD)	3.2 (1.1)	2.9 (1.3)	3.3 (1.0)	4.0 (1.0)	4.1 (0.9)	3.9 (1.1)	2.0 (1.2)	2.4 (1.4)	2.7 (1.3)	3.0 (1.5)

Inter factor correlations of short (20 items) and ultra-short (10 items) versions of BFI are presented in Table 2. Bivariate correlations between factors for short (20 items) and ultra-short (10 items) were presented in the lower and upper diagonal, respectively. At the diagonal are presented the correlations coefficients between the same factors per version. Lastly, normative data is depicted in Table 3 aiming to reflect the typical or average performance in each personality factor in the population under study.

## Discussion

The purpose of this study was to assess the factorial structure of the BFI version featuring 44 items that was translated and validated for the Brazilian population^12^, in addition to assessing the validity and reliability of the short and ultrashort versions of the BFI with 20 and 10 items, respectively. Furthermore, we sought to present normative data to reflect the typical or average performance in the population under study from the short and ultra-short version of the BFI, for the Brazilian population. As for the main outcome, we verified that the short and ultrashort versions of the BFI presented an excellent factorial adjustment.

As for the investigation of the of the psychometric properties of the 44-item version with data from our population, as adapted by Andrade^12^, we were able to identify that there was a poor adaptation of the model. The study carried out by Junior et al.^7^ corroborates this finding, indicating low adaptation levels for the 44-item version. However, these findings are not in accordance with BFI validation studies carried out in other countries, which showed appropriate internal consistency regarding the original model^8–10^.

Similarly, other countries such as France, the United States and Germany tested the short version of the BFI with 10 items, having concluded that the short version maintains significant reliability and validity levels when compared to the original BFI ^13,14^. The study carried out by Junior et al.^7^ also verified better adaptation indexes in the short version of the instrument, however, consisting of 25 items.

In this study, we were able to gather good evidence of validity for the internal structure of the two short versions. The ultrashort version, consisting of 10 items, showed high reliability, which corroborates findings from previous studies ^13,14^. The BFI-10 has been tested in other countries with mixed results^17,18^. Nevertheless, Rammstedt & John^19^, indicated that the ultrashort versions of the BFI maintain significant reliability and validity levels, being seen as appropriate instrument to be used for the purpose of carrying out surveys with limited time. Moreover, we were able to present normative data to reflect the typical or average performance for the Brazilian population of the 20- and 10-item scales with mean, standard deviation, and percentiles.

The validity of the shorter versions of the BFI were deemed appropriate with a bivariate correlation between the five personality factors. Additionally, all analyzed items showed strong correlations between the short versions featuring 20 and 10 items.

As limitations, this study considers smaller versions of the instrument under study. However, it has not conducted all possible comparisons of reduction in the literature. E.g., the BFI-10^19^ has been translated into many different languages and used in a variety of cultural contexts. However, some researchers have questioned the factor structure, suggesting that cultural differences in the meaning and expression of personality traits may not be adequately captured by the BFI-10 in some countries ^20^. In this way, it has been preferred to make a reduction rather than confirming the structure of other reductions in other cultural contexts.

We concluded that the short (20 items) and ultrashort (10 items) of the BFI are reliable to measure personality of the Brazilian population. Both versions of the instrument allow for quick completion, thus increasing its applicability in surveys assessing the personality construct as a secondary instead of a main variable of the study. It must be highlighted that the shorter versions are indicated for use only in a survey context, not being recommended for use in a clinical context.

## Supplementary Information

Supplementary Information.

## Data Availability

The datasets used and/or analysed during the current study available from the prof. Irigaray on reasonable request.
