# Supplementary material for: Evaluating the complete (44-item), short (20-item) and ultra-short (10-item) versions of the Big Five Inventory (BFI) in the Brazilian population
Source: Sci Rep. 2023 May 5;13:7372. doi: 10.1038/s41598-023-34504-1 (PMC10163274; doi:10.1038/s41598-023-34504-1)
Supplement: Supplementary file 1 — Supplementary Information. [file 41598_2023_34504_MOESM1_ESM.docx]

**Attachment A - Big Five Inventory - 10 - BFI-10**

**INSTRUCTIONS**: Below are some characteristics that may or may not apply to you. Please choose a number on the scale below that best expresses your opinion regarding yourself and write it down in the blank space next to each statement. It should be noted that there are no right or wrong answers. Please use the following answer scale:

1 - Completely disagree

2 - Partially disagree

3 - Neither agree nor disagree

4 - Partially agree

5 - Completely agree

I see myself as someone who...

1. Who is talkative, communicative.

2. Likes to cooperate with others.

3. Is original, always having new ideas.

4. Is inventive, creative.

5. Is helpful and assists other people.

6. Does things in an efficient way.

7. Is sociable, extroverted.

8. Is a reliable worker.

9. Gets tense often.

10. Gets nervous easily.

**Attachment B - Big Five Inventory - 20 - BFI-20**

**INSTRUCTIONS:** Below are some characteristics that may or may not apply to you. Please choose a number on the scale below that best expresses your opinion regarding yourself and write it down in the blank space next to each statement. It should be noted that there are no right or wrong answers. Please use the following answer scale:

1 - Completely disagree

2 - Partially disagree

3 - Neither agree nor disagree

4 - Partially agree

5 - Completely agree

I see myself as someone who...

1. Who is talkative, communicative.

2. Keeps going until the task or work is completed.

3. Is depressed, sad.

4. Likes to cooperate with others.

5. Is original, always having new ideas.

6. Is inventive, creative.

7. Is emotionally stable, does not get upset easily.

8. Is helpful and assists other people.

9. Is kind, considerate of others.

10. Tends to be lazy.

11. Does things in an efficient way.

12. Is sociable, extroverted.

13. Is reliable in general.

14. Is full of energy.

15. Is a reliable worker.

16. Has a fertile imagination.

17. Gets tense often.

18. Gets nervous easily.

19. Likes to reflect on things, play with ideas.

20. Tends to be quiet, silent..
